# Heterogenous Induction of Blocking Antibodies against Ragweed Allergen Molecules by Allergen Extract-Based Immunotherapy Vaccines

**DOI:** 10.3390/vaccines12060635

**Published:** 2024-06-07

**Authors:** Lauriana-Eunice Zbîrcea, Maria-Roxana Buzan, Manuela Grijincu, Monica-Daniela Cotarcă, Tudor-Paul Tamaș, Laura Haidar, Gabriela Tănasie, Ioan Huțu, Elijahu Babaev, Frank Stolz, Rudolf Valenta, Virgil Păunescu, Carmen Panaitescu, Kuan-Wei Chen

**Affiliations:** 1 Center of Immuno-Physiology and Biotechnologies, Department of Functional Sciences, Victor Babes University of Medicine and Pharmacy, 300041 Timisoara, Romaniahaidar.laura@umft.ro (L.H.);; 2 OncoGen Center, Pius Brinzeu County Clinical Emergency Hospital, 300723 Timisoara, Romania; 3 Faculty of Veterinary Medicine, University of Life Sciences “King Mihai I of Romania”, 300645 Timișoara, Romania; ioan.hutu@fmvt.ro; 4 Vienna Competence Center, Biomay AG, 1090 Vienna, Austria; 5 Center of Pathophysiology, Infectiology and Immunology, Department of Pathophysiology and Allergy Research, Division of Immunopathology, Medical University of Vienna, 1090 Vienna, Austria; 6 Laboratory for Immunopathology, Department of Clinical Immunology and Allergology, Sechenov First Moscow State Medical University, 119991 Moscow, Russia; 7 Karl Landsteiner University of Health Sciences, 3500 Krems, Austria; 8 NRC Institute of Immunology FMBA of Russia, 115478 Moscow, Russia

**Keywords:** ragweed allergy, allergen-specific immunotherapy (AIT), allergen-specific IgG, IgE inhibition

## Abstract

Currently, allergen-specific immunotherapy (AIT) for ragweed allergy is still based on natural allergen extracts. This study aimed to analyse the ability of four commercially available AIT vaccines (CLUSTOID, TYRO-SIT, POLLINEX Quattro Plus and Diater Depot) regarding their ability to induce IgG antibodies against ragweed pollen allergens in rabbits. Accordingly, the IgG reactivity of AIT-induced rabbit sera was tested for ten different ragweed pollen allergens (Amb a 1, 3, 4, 5, 6, 8, 9, 10, 11 and 12) by an ELISA. Furthermore, the ability of rabbit AIT-specific sera to block allergic patients’ IgE binding to relevant ragweed allergens (Amb a 1, 4, 6, 8 and 11) and to inhibit allergen-induced basophil activation was evaluated by an IgE inhibition ELISA and a mediator release assay. Only two AIT vaccines (Diater Depot > CLUSTOID) induced relevant IgG antibody levels to the major ragweed allergen Amb a 1. The IgG responses induced by the AIT vaccines against the other ragweed allergens were low and highly heterogeneous. Interestingly, the kinetics of IgG responses were different among the AIT vaccines and even within one AIT vaccine (Diater Depot) for Amb a 1 (long-lasting) versus Amb a 8 and Amb a 11 (short-lived). This could be due to variations in allergen contents, the immunogenicity of the allergens, and different immunization protocols. The IgE inhibition experiments showed that rabbit AIT-specific sera containing high allergen-specific IgG levels were able to inhibit patients’ IgE binding and prevent the mediator release with Diater Depot. The high levels of allergen-specific IgG levels were associated with their ability to prevent the recognition of allergens by patients’ IgE and allergen-induced basophil activation, indicating that the measurement of allergen-induced IgG could be a useful surrogate marker for the immunological efficacy of vaccines. Accordingly, the results of our study may be helpful for the selection of personalized AIT vaccination strategies for ragweed-allergic patients.

## 1. Introduction

*Ambrosia artemisiifolia* (common or short ragweed) pollen is an important allergen source able to induce allergic manifestations in late summer and fall with a high impact on public health [1,2,3]. So far, eleven ragweed pollen allergens have been identified, and among them, two have been described as major allergens: Amb a 1 and Amb a 11 [4,5,6]. The major allergen Amb a 1 belongs to the family of pectate lyases, it has five isoforms and is known as the most important allergen in the ragweed pollen with a greater than 90% IgE sensitization rate in ragweed-allergic patients [7,8,9]. More recent studies also consider cysteine protease Amb a 11 to be a major ragweed pollen allergen with an IgE sensitization rate of up to 68% [10,11]. Furthermore, some less frequently recognized allergens are considered relevant, such as defensin Amb a 4, non-specific lipid transfer protein Amb a 6 and profilin Amb a 8 [12] due to their ability to cross-react to other homologous allergens, such as Amb a 4 with Art v 1 [13,14], Amb a 6 with Art v 3 [15] and Amb a 8 with several profilins [16,17,18]. Other allergens, such as plastocyanin Amb 3, Amb a 5 (unknown function), calcium-binding proteins Amb a 9 and Amb a 10, and the enolase Amb a 12, may also contribute to the IgE sensitization of ragweed-allergic patients [19,20,21,22].

Exposure to ragweed pollen may trigger symptoms of rhinitis and conjunctivitis which can progress towards severe asthma-like symptoms, significantly impacting the quality of life of sensitized individuals [23,24,25]. Currently, allergen-specific immunotherapy (AIT) is the only form of allergen-specific treatment able to modify the course of the disease and prevent the progression of rhinitis towards asthma [26,27,28]. AIT is based on repeated immunizations (i.e., vaccinations) with the disease-causing allergen to induce a protective allergen-specific response and to downregulate allergen-specific inflammatory cell responses [29,30]. The fact that even passive immunization with allergen-specific IgG antibodies, which block allergic patients’ IgE binding to allergens, strongly reduces allergen-specific inflammation has shown blocking IgG antibodies to be a major mechanism within successful AIT [31,32]. Subcutaneous vaccination is the oldest and the most studied form of therapy for allergy treatment, but in recent years, sublingual immunotherapy (SLIT) consisting of tablets or drops that contain small allergen amounts has also become a common form of treatment for patients with seasonal allergies [33,34,35].

Nowadays, ragweed allergy AITs are still formulated using natural allergen extracts [36,37]. The majority of the commercially available ragweed AITs are based on natural unmodified pollen extract, while in allergoids, the extracts undergo allergen-modifying processes to decrease allergenic activity and maintain immunogenicity, i.e., the ability to induce allergen-specific blocking IgG [38,39]. POLLINEX Quattro Ragweed is an allergoid with reported safety in animals and efficacy in reducing allergy symptoms and is commonly used for ragweed allergy treatment [40,41]. However, the main problem with these pollen-based AITs is the various allergen contents and concentrations, which can vary greatly between different extracts and even more from batch to batch [42,43]. Furthermore, the reduction in the allergenic activity in allergoids may affect their ability to induce blocking IgG antibodies [44,45]. Variations in the allergen content may be due to different methods of extraction and processing, which may further affect the immunogenicity of individual allergens [46]. The ability of AIT vaccines to induce IgG antibodies capable of inhibiting allergic patients’ IgE binding to allergens can be investigated by the immunization of animals and testing of the IgE blocking capacity of the induced antibodies [47,48]. This investigation can be easily carried out with subcutaneous vaccines, whereas testing SLIT may prove challenging for animal models.

This study aimed to investigate the ability of different commercially available ragweed pollen extract-based subcutaneous AIT vaccines to induce allergen-specific IgG antibodies in rabbits and to further characterize the blocking abilities of these induced IgG antibodies. Therefore, four commercially available ragweed pollen-based AITs were selected for rabbit immunization, and rabbit AIT-specific sera were used to identify the allergen-specific IgG response towards the ragweed extract and a comprehensive panel of ragweed pollen allergen molecules, i.e., Amb a 1, Amb a 3, Amb a 4, Amb a 5, Amb a 6, Amb a 8, Amb a 9, Amb a 10, Amb a 11 and Amb a 12. The ability of the allergen-specific IgG antibodies to inhibit patients’ IgE binding and allergen-induced mediator release was also evaluated. The results from our study could help to direct the allergist in the prescription of personalized AIT vaccination for the treatment of ragweed pollen allergies based on patients’ IgE sensitization profiles.

## 2. Materials and Methods

### 2.1. Expression and Purification of the Recombinant Allergens and Extract Preparation

Genes coding for Amb a 1.0101, Amb a 3.0101, Amb a 4.0101, Amb a 5.0101, Amb a 6.0101, Amb a 8.0101, Amb a 9.0101, Amb a 10.0101, Amb a 11.0101 and Amb a 12.0102 (sequences available on WHO/IUIS Allergen Nomenclature (www.allergen.org (accessed on 15 April 2024)) were synthesized and codon-optimized for *Escherichia coli* or *Spodoptera frugiperda* insect cell expression. Recombinant (r) Amb a 1.01, rAmb a 3, rAmb a 4, rAmb a 6, rAmb a 11 and rAmb a 12 were produced in insect cells similarly to the process described in [49]. rAmb a 5, rAmb a 8, rAmb a 9 and rAmb a 10 were expressed in *E. coli* as described in [21]. Purified allergens were further stored at −20 °C.

Aqueous ragweed pollen extract was prepared by mixing 2 g of ragweed (*Ambrosia artemisiifolia*; Allergon AB, Ängelholm, Sweden) pollen with 20 mL of sterile Dulbecco’s phosphate-buffered saline (DPBS, Gibco, Thermo Fisher Scientific, Waltham, MA, USA) and stirred for 4 h at room temperature. The pollen was then centrifuged (20,000× *g*, 30 min, 4 °C) and the supernatant was dialyzed against fresh DPBS overnight at 4 °C. The obtained extract was then stored at −20 °C until use.

### 2.2. Sera from Allergic Patients

Fifty ragweed-allergic patients were included in this study based on a documented case history of ragweed allergy and a positive skin prick test [12]. Written consent was obtained from all patients before blood sample collection. Serum samples were tested in ELISA to confirm IgE reactivity to at least one of the following ragweed pollen allergens: rAmb a 1.01, rAmb a 4, rAmb a 6, rAmb a 8 or rAmb a 11 (Table S1). Sera were stored at −80 °C until use. The study was approved by the local ethics committee of the County Emergency Clinical Hospital “Pius Brînzeu”, Timisoara.

### 2.3. Subcutaneous Allergen-Specific Immunotherapy Vaccines for the Treatment of Ragweed Pollen Allergy

Ragweed SCIT products were purchased from four companies. The following products were used: CLUSTOID Cluster-Allergoid W301 (ragweed 100%; reference no. 7049363; expiration date, October 2020, ROXALL Medizin, Vienna, Austria); TYRO-SIT (ragweed 100%; batch: 293657, reference no. 2019/050/020557, expiration date, October 2020, Bencard Allergie GmbH, Munich, Germany); POLLINEX Quattro Plus (ragweed 100%; batch: 293693; reference no. 2019/050/020558; expiration date, October 2020, Bencard Allergie GmbH, Munich, Germany); and Diater Depot (Ambrosia 100%; reference no. RO00000207; expiration date, December 2020; DIATER, Madrid, Spain).

CLUSTOID is based on an allergoid made from ragweed extract and absorbed to aluminium hydroxide. TYRO-SIT and POLLINEX [40] are based on modified ragweed allergen extracts and absorbed onto L-Tyrosin; additionally, POLLINEX contains the immunostimulatory adjuvant monophosphoryl lipid A. Diater is based on ragweed allergen extract and absorbed onto aluminium hydroxide. The AIT products were stored as recommended by the manufacturers (2–8 °C) and used for rabbit immunization exactly according to the manufacturer’s recommendations before their expiration dates.

### 2.4. Immunization of Rabbits with AIT Vaccines and Purified Recombinant Ragweed Pollen Allergens

**Figure 1:**
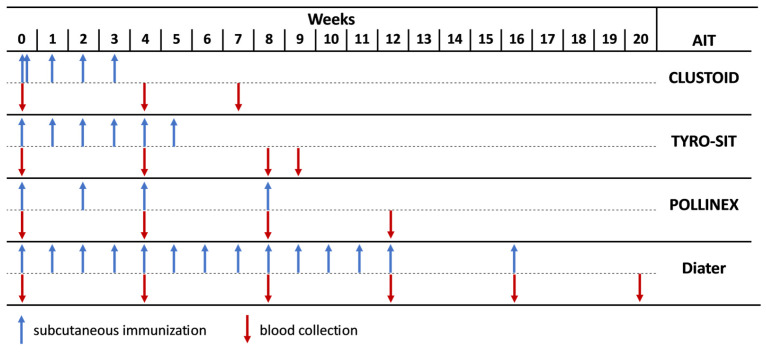
Scheme of rabbit immunizations with different ragweed pollen AIT vaccines. Rabbits were immunized with the registered AIT vaccines, CLUSTOID, TYRO-SIT, POLLINEX and Diater according to the manufacturer’s recommendation. Serum samples were collected from all rabbits before immunization (PIS) and every four weeks (IS), and final serum samples (final IS) were collected four weeks after the last immunization, e.g., for TYRO-SIT, PIS was collected before immunization (week 0), IS was collected every four weeks (week 4 and week 8) and final IS was collected 4 weeks after the last immunization (week 9).

New Zealand White (NZW) rabbits (*n* = 2 rabbits per vaccine) were immunized subcutaneously (Davids Biotechnologie GmbH, Regensburg, Germany) with the ragweed AIT vaccines from different companies (CLUSTOID, ROXALL Medizin; TYRO-SIT and POLLINEX Quattro Plus, Bencard Allergie GmbH; Diater Depot, DIATER) as recommended by the manufacturer for AIT treatment in patients (Table S2). For CLUSTOID, the shortened initial schedule of the immunotherapy was applied, consisting of 2 injections of increasing doses administered on the first day, and 3 immunizations of the same doses were administered in weekly intervals (Figure 1, Table S2). For TYRO-SIT, 6 injections of increasing doses were administered in weekly intervals, and for POLLINEX, 3 injections were administered with a 2-week interval followed by a fourth injection after 4 more weeks (Figure 1, Table S2). In the case of Diater, 13 injections of increasing doses were administered in weekly intervals and another one 4 weeks after the 13th vaccine (Figure 1, Table S2). The treatment was completed for CLUSTOID in 3 weeks, for TYRO-SIT in 5 weeks, for POLLINEX in 8 weeks and for Diater in 16 weeks. Serum samples were obtained from the rabbits before the first immunization (pre-immune sera, PIS) and every 4 weeks during immunization (immune sera, IS), and the final bleeding was taken 4 weeks after the last injection (final IS).

For control purposes, antisera were raised against individual recombinant ragweed allergen molecules. For this purpose, NZW rabbits were injected with 200 µg of the recombinant allergens (rAmb a 1.01, rAmb a 4, rAmb a 6 and rAmb a 8) three times using Freund’s complete adjuvant once and Freund’s incomplete adjuvants twice in monthly intervals. The final serum was collected 10 days after the last immunization, and the IgG titre was determined by ELISA. The animal study protocol for the allergen molecules was approved by the Ethics Committee of University of Agricultural Sciences and Veterinary Medicine “King Michael I of Romania” in Timisoara.

### 2.5. Measurement of Ragweed Pollen Allergen-Specific Antibodies by ELISA

Recombinant allergens from ragweed (Amb a 1.01, Amb a 3, Amb a 4, Amb a 5, Amb a 6, Amb a 8, Amb a 9, Amb a 10, Amb a 11 and Amb a 12) were coated on Nunc MaxiSorp ELISA plates (0.5 µg per well in PBS; Thermo Fisher Scientific, Dreieich, Germany) overnight at 4 °C. Natural (n) Amb a 1.01 and rAmb a 1.03 obtained from Biomay AG (Vienna, Austria) were used along with rAmb a 1.01 for comparison. Plates were washed two times with PBS 0.05% (*v*/*v*) Tween 20 (PBST) and blocked with PBST containing 3% BSA (*w*/*v*) for 2.5 h at room temperature. Plates were incubated with the rabbits’ PIS and antisera diluted 1:1000 in PBST 0.5% (*w*/*v*) BSA overnight at 4 °C. After washing the plates with PBST 5 times, bound rabbit IgG antibodies were detected with 100 µL/well of 1:2000-diluted donkey anti-rabbit IgG antibodies labelled with horseradish peroxidase (HRP) (GE Healthcare UK Limited, Glasgow, UK) for 45 min at 37 °C and 45 min at 4 °C. Colour development was achieved using staining solution of 2,2-azino-bis (3-ethylbenzthiazoline-6-sulfonic acid) diammonium salt (ABTS, 100 µL/well, Sigma Aldrich, St. Louis, MO, USA). Optical density (OD) was measured at 405 nm after 30 min. The rabbit immunized with each AIT showing the highest IgG titre to the tested allergens was further tested in titration ELISA as described above with the following dilutions: 1:100, 1:500, 1:1000, 1:5000, 1:10,000 and 1:50,000. Measurements were carried out as duplicates with a deviation of less than 10%, and the results are shown as mean OD values.

### 2.6. Inhibition of Human IgE Binding to Recombinant Ragweed Pollen Allergens by AIT Vaccine-Induced IgG Antibodies in ELISA Competition Assays

ELISA plates were coated with 50 µg/mL of ragweed pollen extract and 5 µg/mL of each of the allergens, i.e., rAmb a 1, rAmb a 4, rAmb a 6, rAmb a 8 and rAmb a 11, and incubated overnight at 4 °C. The plates coated with the extract were incubated with 1:10 diluted rabbit final IS and with 1:10 diluted rabbit PIS for control purposes overnight at 4 °C. The plates coated with the allergens were incubated with 1:5 diluted rabbit final IS and with 1:5 diluted rabbit PIS, respectively. Additional 1:5 diluted rabbit IS collected at different time points was used in the case of CLUSTOID (week 4) and Diater (weeks 4, 8 and 12) for the allergens whose allergen-specific IgG titre in IS was higher compared to the final IS. After washing, the plates were incubated with 1:5 diluted sera from patients with ragweed allergy. Bound IgE antibodies were detected with HRP-coupled goat anti-human IgE antibodies diluted to 1:2500 (Sera Care, Milford, MA, USA) for 45 min at 37 °C and 45 min at 4 °C. Colour development and measurements were performed as described above. Measurements were carried out for each patient as duplicates with a deviation of less than 10% and shown as mean OD values (Table S3). The same rabbit serum used in the titration experiments was also used for the inhibition experiments for each allergen. Patients with specific IgE reactivity (OD values ≥ 0.4) were included in this study. The percentage of inhibition of IgE binding was calculated using extinctions measured after preincubation with rabbit immune (ODI) and pre-immune (ODP) sera as follows:

IgE binding inhibition percentage = (1 − ODI/ODP) × 100

### 2.7. Inhibition of Allergen-Induced Basophil Activation by AIT-Specific IgG Antibodies in Rat Basophil Leukaemia (RBL) Assays

To investigate the ability of rabbit antisera to inhibit allergen-induced basophil degranulation, human serum IgE (diluted 1:10) from a total of 16 ragweed-allergic patients who tested positive to the allergens rAmb a 1.01, rAmb a 4, rAmb a 6 rAmb a 8 and Amb a 11 (Table S4) were loaded to huRBL RS-ATL8 cells. Then, rabbit sera (the same rabbit IS used in the competition ELISA) that were diluted to 1:10 were preincubated with three different allergen concentrations (100, 10 and 1 ng/mL) as described and added to the IgE-loaded basophils [50]. Measurements were carried out in triplicates, and results are expressed as a percentage of the maximum β-hexosaminidase release (complete cell lysis by 1% Triton X-100), and the inhibition percentage of the β-hexosaminidase release was calculated according to the following formula [50]:

[((R_P_ − R_BP_) − (R_I_ − R_BI_))/(R_P_ − R_BP_) × 100]

where R_P_ = β-hexosaminidase release when RBL cells were incubated with patient sera and ragweed allergen pre-incubated with pre-immune rabbit sera; R_BP_ = β-hexosaminidase release when RBL cells were incubated with ragweed allergen pre-incubated with pre-immune rabbit sera; R_I_ = β-hexosaminidase release when RBL cells were incubated with the patient serum and ragweed allergen pre-incubated with rabbit anti-CLUSTOID, -TYRO-SIT, -POLLINEX, -Diater IS or final IS or with allergen-specific serum; and R_BI_ = β-hexosaminidase release when RBL cells were incubated with ragweed allergen pre-incubated with rabbit anti-CLUSTOID, -TYRO-SIT, -POLLINEX, -Diater IS or final IS or allergen-specific serum.

## 3. Results

### 3.1. Only Rabbits Immunized with CLUSTOID and Diater Had Relevant Anti-Ragweed Pollen Extract-Specific IgG Responses Four Weeks after the Last Injection

The ragweed allergen extract-specific IgG levels of rabbits immunized with CLUSTOID, TYRO-SIT, POLLINEX and Diater were evaluated by testing the rabbits’ pre-immune, immune and final immune sera against the ragweed pollen extract and the individual ragweed pollen allergens. The rabbits were immunized according to the manufacturer’s recommendation to be used for AIT in allergic patients (Table S2), and their immune sera were collected at different time points, as shown in Figure 1.

**Figure 2:**
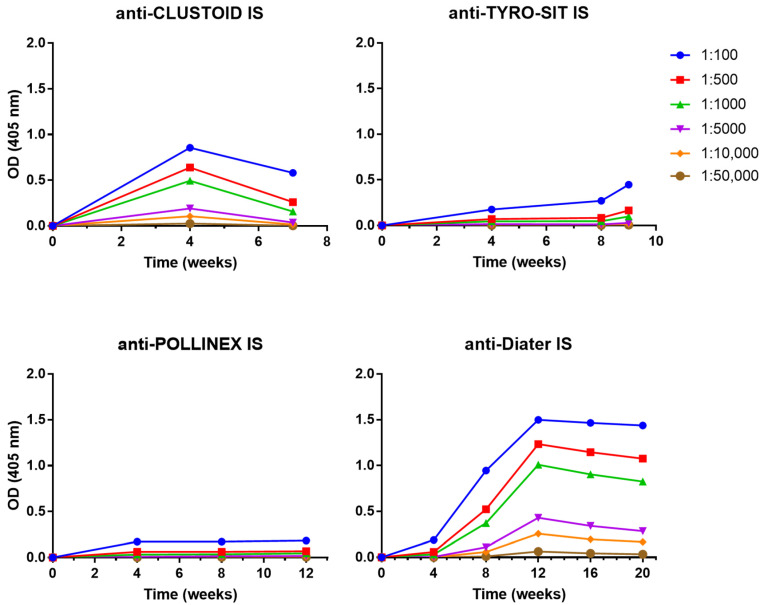
Induction of ragweed pollen allergen-specific IgG responses in rabbits by immunization with CLUSTOID, TYRO-SIT, POLLINEX and Diater. For CLUSTOID, 5 injections were administered over 3 weeks, for TYRO-SIT, 6 injections were administered in 5 weeks, for POLLINEX, 4 injections were administered in 8 weeks and for Diater 14 injections were administered in 16 weeks. Rabbit sera were collected before immunization (week 0) and every four weeks (i.e., week 4 for CLUSTOID, weeks 4 and 8 for TYRO-SIT and POLLINEX and weeks 4, 8, 12 and 16 for Diater) until final serum collection four weeks after the last immunization (i.e., week 7 for CLUSTOID, week 9 for TYRO-SIT, week 12 for POLLINEX and week 20 for Diater). Rabbit sera were diluted from 1:100 down to 1:50,000 and tested against ragweed pollen extract in ELISA. Mean OD values corresponding to allergen-specific IgG levels (*y*-axes) at different time points (*x*-axes) are shown. IS, immune serum.

Different dilutions of the sera from the rabbits immunized with the four AIT vaccines were prepared to determine their IgG levels during the course of the immunotherapy. In order to obtain comparable results for the four AIT vaccines, serum samples were not only obtained in monthly intervals but also 4 weeks after the last immunization (Figure 1). The results showed that Diater induced the highest IgG levels against the ragweed extract at week 12 (OD = 1.49) with the rabbit IS diluted to 1:100, and the IgG levels remained elevated one month after the end of the treatment (OD = 1.43 at week 20) with specific IgG binding detected down to the level of 1:10,000 diluted rabbit IS (Figure 2). Upon immunization with CLUSTOID, ragweed pollen extract-specific IgG levels peak at week 4 (OD = 0.85) with the rabbit IS diluted to 1:100, and the IgG response was still detected down to the level of 1:10,000. However, the ragweed extract-specific IgG decreased strongly already one month after the last injection in serum diluted to 1:100 (OD = 0.58 at week 7) with a signal detectable only until a dilution of 1:5000 (Figure 2). The TYRO-SIT-immunized rabbits showed almost no IgG reactivity to the ragweed pollen extract. Only one month after the end of the immunization (week 9), specific IgG levels were detected when the serum was diluted to 1:100 (OD = 0.44) (Figure 2). The POLLINEX-immunized rabbits showed the lowest IgG response towards the extract with specific IgG detected only at a dilution of 1:100 with constantly very low IgG levels (OD ~ 0.17) from week 4 to week 12 (one month after the end of the immunization) (Figure 2). Comparable but not completely identical results were obtained for each of the two rabbits immunized with a given AIT vaccine (Figure S1).

### 3.2. Levels and Kinetics of IgG against rAmb a 1.01, nAmb a 1.01 and rAmb a 1.03 in AIT Vaccine-Immunized Rabbits

**Figure 3:**
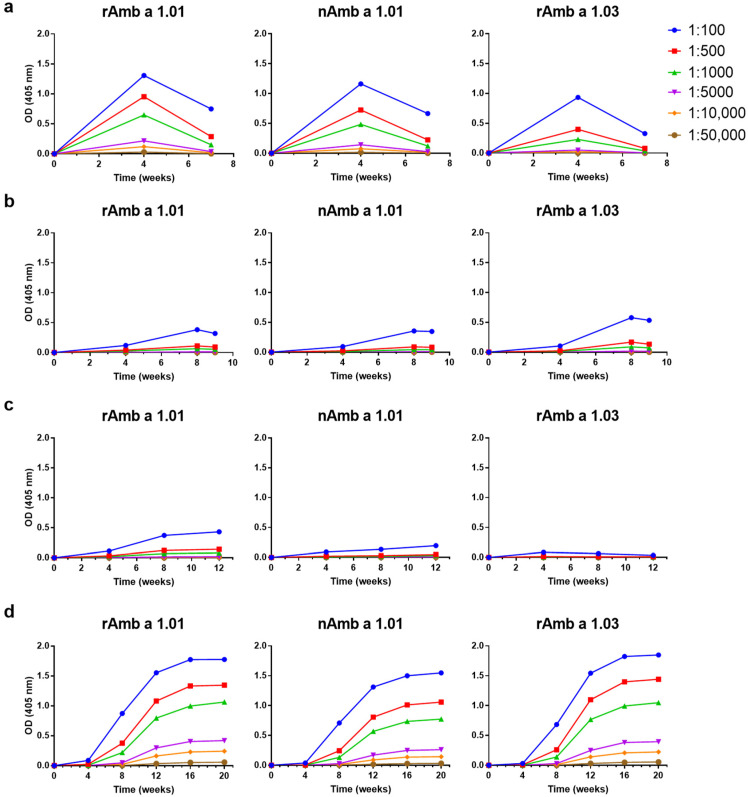
Comparison of levels and time courses of allergen-specific IgG responses against rAmb a 1.01, nAmb a 1.01 and rAmb a 1.03 upon immunization with (**a**) CLUSTOID, (**b**) TYRO-SIT, (**c**) POLLINEX and (**d**) Diater. Sera from rabbits immunized with the four AITs were collected before immunization and every four weeks until final serum collection four weeks after the last immunization. Rabbit pre-immune and immune serum was titrated starting at 1:100 down to 1:50,000. The mean OD values corresponding to allergen-specific IgG levels (*y*-axes) at different time points (*x*-axes) are shown.

The IgG responses towards rAmb a 1.01, nAmb a 1.01 and rAmb a 1.03 of the rabbits immunized with the four AIT vaccine-specific sera are displayed in Figure 3. The levels and kinetics of the Amb a 1-specific IgG antibodies were similar to those against the ragweed extract (Figure 2) in terms of increases and decreases for Diater and CLUSTOID. The highest Amb a 1-specific IgG levels were induced by Diater at week 20 (OD = 1.77) and by CLUSTOID at week 4 (OD = 1.30) when the sera were diluted to 1:100 with signals detected down to a dilution of 1:10,000 (Figure 3a,d). Specific IgG levels were also found in rabbits immunized with TYRO-SIT and POLLINEX but these were very low (Figure 3b,c). No relevant IgG towards rAmb a 1.03 was detectable in the rabbits immunized with POLLINEX (Figure 3c). Interestingly, rAmb a 1.03-specific IgG levels were comparable to rAmb a 1.01-specific IgG for Diater, lower for CLUSTOID and POLLINEX and higher for TYRO-SIT, respectively (Figure 3a–d). The kinetics of the Amb a 1-specific antibody responses were similar to those against the allergen extracts. For CLUSTOID, the Amb a 1-specific IgG dropped strongly 4 weeks after the last immunization, whereas for Diater, TYRO-SIT and POLLINEX, the IgG levels did not show such a strong decrease (Figure 3a–d). If comparing rAmb a 1-specific IgG levels between AITs, a similar IgG response was found for TYRO-SIT and POLLINEX throughout the immunization (Figure 3b,c). If comparing the IgG response to different time points, similar responses were found for TYRO-SIT, POLLINEX and DIATER only at week 4 (Figure 3b–d). Thus, it seemed that there is a heterogeneity of Amb a 1-isoform-specific IgG induction by the different AIT vaccines.

### 3.3. Levels and Kinetics of Allergen-Specific IgG Responses Induced by the Four AIT Vaccines to Ragweed Pollen Allergen Molecules Vary

**Figure 4:**
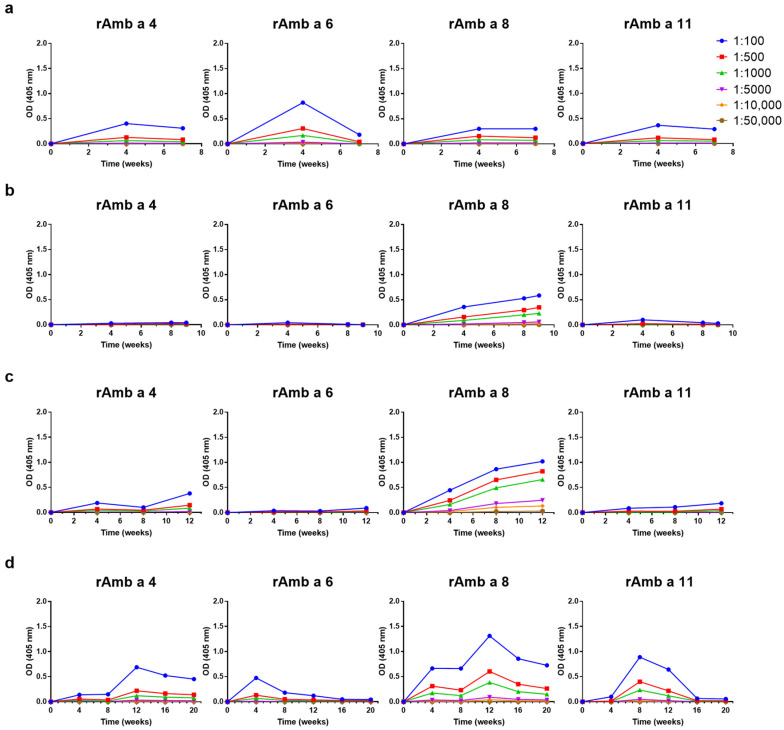
Comparisons and time courses of allergen-specific IgG responses of rabbits against Amb a 4, Amb a 6, Amb a 8 and Amb a 11 upon immunization with (**a**) CLUSTOID, (**b**) TYRO-SIT, (**c**) POLLINEX and (**d**) Diater. Sera from rabbits immunized with the four AITs were collected before immunization and every four weeks until final serum collection four weeks after the last immunization. Rabbit pre-immune and immune serum was titrated starting at 1:100 down to 1:50,000. Mean OD values corresponding to allergen-specific IgG levels (*y*-axes) at different time points (*x*-axes) are shown.

Next, we investigated the levels and kinetics of the IgG responses induced by the four AIT vaccines against recombinant Amb a 3, Amb a 4, Amb a 5, Amb 6, Amb 8, Amb a 9, Amb a 10, Amb a 11 and Amb a 12. CLUSTOID induced IgG antibodies against Amb a 4, Amb a 6, Amb a 8, Amb a 10, Amb a 11 and Amb a 12, whereas low or no relevant IgG responses were induced against Amb a 3, Amb a 5 and Amb a 9 (Figure 4a and Figure S2a). There were distinct IgG responses to Amb 5, Amb a 8, Amb a 9 and Amb a 12, but no or very low IgG responses to Amb a 3, Amb a 4, Amb a 6, Amb a 10 and Amb a 11 were found upon immunization with TYRO-SIT (Figure 4b and Figure S2b).

POLLINEX induced IgG responses against Amb a 4, Amb a 5, Amb a 8, Amb a 9, Amb a 10 and Amb a 12 but not strong responses against Amb a 3, Amb a 6 or Amb a 11 (Figure 4c and Figure S2c). For Diater, induced IgG responses against Amb a 4, Amb a 5, Amb a 6, Amb a 8, Amb a 9, Amb a 10, Amb a 11 and Amb a 12 were found but not against Amb a 3 (Figure 4d and Figure S2d). It was interesting to note that the kinetics of the IgG responses to Amb a 1 and the other ragweed pollen allergen molecules were different. Especially for Diater, the level of Amb a 1-specific IgG remained high 4 weeks after the last injection, whereas for Amb a 6, Amb a 9, Amb a 10 and Amb a 11, the level of IgG increased transiently at the beginning of the immunization and then disappeared and the level of IgG specifically for Amb a 4, Amb a 8 and Amb a 12 decreased quite strongly 4 weeks after the last immunization (Figure 4d and Figure S2d). For Amb a 5, the kinetics in Diater were similar to those of Amb a 1 (Figure S2d). Similar increasing and decreasing trends in IgG levels can be seen in the case of Amb a 4 for POLLINEX and Diater up to week 12, in the case of Amb a 6 for CLUSTOID and Diater up to week 8 and in the case of Amb a 8 for TYRO-SIT, POLLINEX and Diater up to week 12 (Figure 4). Our results thus show that AIT vaccines based on natural allergen extracts induce different IgG responses against the individual allergen molecules, which even varied in the two rabbits tested for each of the AIT vaccines (Figure S3). Moreover, even within one AIT vaccine, the kinetics of IgG responses towards different allergen molecules vary.

### 3.4. Highly Variable Inhibition of Patient’s IgE Binding to Ragweed Pollen Extract and Ragweed Pollen Allergens by AIT Vaccine-Induced Antibodies

**Figure 5:**
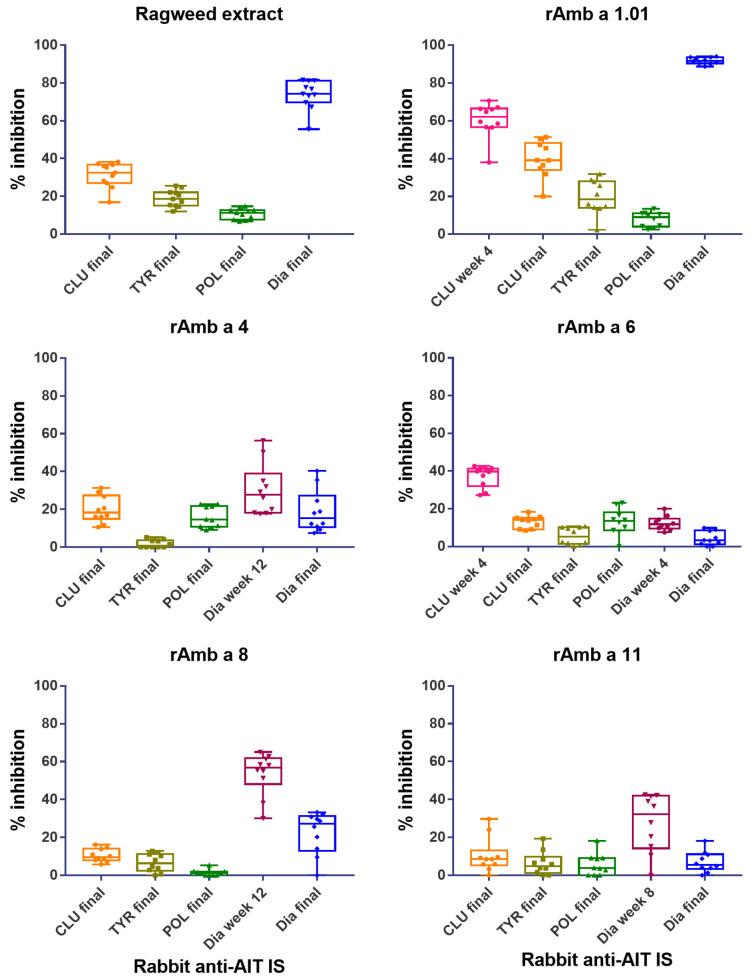
Inhibition of patients’ IgE binding to ragweed pollen extract, rAmb a 1.01, rAmb a 4, rAmb a 6, rAmb a 8 and rAmb a 11 using rabbit anti-CLUSTOID (CLU), -TYRO-SIT (TYR), -POLLINEX (POL) and Diater (Dia) immune sera (IS) and final IS (*x*-axis). Inhibition was performed for eleven patients to ragweed pollen extract and ten patients to each recombinant allergen. Percentages of inhibition of patients’ IgE binding to ragweed extract and allergens are shown on the *y*-axis (boxes represent the 25th and 75th percentile, whiskers show minimum and maximum values and the median is represented by horizontal bars).

The IgE reactivity profiles of the ragweed-allergic patients to the individual ragweed pollen allergens were determined for a comprehensive panel of allergens, including Amb a 1 isoforms, Amb a 3, Amb a 4, Amb a 5, Amb a 6, Amb a 8, Amb a 9, Amb a 10, Amb a 11 and Amb a 12 (Table S1). The sera available from the patients with defined IgE reactivity profiles allowed us to test the ability of the antibodies induced by the four AIT vaccines to inhibit the allergic patients’ IgE binding to defined allergen molecules. As a first step, we evaluated the ability of rabbit IgG antibodies to block IgE binding to ragweed pollen allergen extract (Figure 5, Table S3). For the inhibition experiments, we used rabbit IS from the time point of bleeding with the highest allergen-specific IgG levels as well as the serum from the final bleeding 4 weeks after the last immunization.

Figure 5 shows that the inhibition of the patients’ (*n* = 11) IgE binding to ragweed extract was the strongest and very consistent for the Diater-induced IgG antibodies. In fact, the rabbit anti-Diater antibodies in the final IS showed a mean inhibition of patients’ IgE binding of 73.9 ± 7.7% (mean inhibition ± SD; range: 55.5–81.6%) (Figure 5, Table S3). Much lower inhibitions of IgE binding to ragweed pollen extract were observed for the antibodies induced by the other AIT vaccines, i.e., rabbit anti-CLUSTOID showed an inhibition of 31.3 ± 6.6% (range: 16.9–38.3%); anti-TYRO-SIT showed an inhibition of 18.9 ± 4.4% (range: 12–25.5%) and rabbit anti-POLLINEX showed an inhibition of 10.7 ± 2.8% (range: 6.7–14.7%) (Figure 5; Table S3).

The rabbit anti-Diater antibodies also showed the highest IgE blocking activity towards rAmb a 1.01 in the tested patients (*n* = 10; Table S3) with a mean inhibition value of 91.8 ± 1.7% (range: 88.7–94%) (Figure 5 and Table S3). The rabbit anti-CLUSTOID antibodies obtained at 4 weeks had a mean inhibition of 60.4 ± 9.3% (range: 38–70.6%), but the level of inhibition dropped in the antiserum obtained four weeks after the last immunization to a mean inhibition of only 39.6 ± 9.6% (range: 20–51.4%) (Figure 5, Table S3). Very low inhibitions of IgE binding to Amb a 1 were observed for the rabbit anti-TYRO-SIT antibodies with a mean inhibition value of 19.6 ± 9.1%; (range: 2.2–31.8%) and the rabbit anti-POLLINEX antibodies with a mean inhibition value of 7.9 ± 4.0% (range: 2.5–13.5%) (Figure 5 and Table S3).

We also studied the inhibition of patients’ IgE binding to other frequently recognized allergens (i.e., Amb a 4, Amb a 6, Amb a 8 and Amb a 11) (Table S1). Except for Amb a 6, the rabbit anti-Diater antibodies inhibited patients’ IgE binding better than the antibodies induced by the other AIT vaccines (Figure 5, Table S3). However, the level of inhibition of IgE binding was relatively low. Regarding Amb a 4, the mean inhibition of IgE binding with the serum obtained at week 12 was 30.4 ± 13.7% (range: 18–56.4%), and with the anti-Diater serum obtained 4 weeks after the last immunization, the mean inhibition was 19.0 ± 11.2% (range: 7.5–40.4%) (Figure 5 and Table S3). Thus, the inhibition rates corresponded to Amb a 4-specific antibody levels (Figure 4).

For Amb a 8, rabbit anti-Diater IS obtained at week 12 yielded a mean level of inhibition of IgE binding of 53.6 ± 11.2% (range: 30.1–65%), which dropped 4 weeks after the last immunization to a mean inhibition of 22.5 ± 11.2% (range: 0–33.3%) (Figure 5 and Table S3). The Amb a 11 rabbit anti-Diater antibodies obtained at week 8 showed a mean inhibition value of 29.1 ± 13.0% (range: 10.6–42.5%) (Table S3), and, in agreement with the antibody levels (Figure 4), the rabbit anti-Diater final IS mean level of inhibition of IgE binding declined to 6.4 ± 5.9% (range: 0–18.1%). The antibody levels specific for Amb a 6 after Diater immunization were low (Figure 4), and accordingly, the level of inhibition of IgE binding was very low (i.e., below 20%) (Figure 5). Only CLUSTOID induced relevant Amb a 6-specific IgG responses (Figure 4), and anti-CLUSTOID antibodies were the only ones to inhibit IgE binding to Amb a 6 (Figure 5), whereas the other AIT vaccines did not induce IgE-blocking antibodies for Amb a 6 in a relevant manner (Figure 5, Table S3).

### 3.5. AIT Vaccine-Induced Antibodies Block Allergen-Induced Basophil Degranulation to a Varying Degree

The use of basophils loaded with serum IgE from allergic patients is a well established surrogate model for measuring the ability of protective antibodies to inhibit an immediate allergic reaction. This in vitro model has been developed for measuring protective antibodies that develop in the course of AIT originally with allergic patients’ basophils [51]. It has been further refined by using rat basophils expressing the human high-affinity receptor for IgE, huFcεRI [52]. In order to study the ability of AIT vaccine-induced antibodies to inhibit allergen-specific basophil activation, we loaded rat basophils expressing huFcεRI with serum IgE from patients and then exposed the cells to allergens in the presence or absence of AIT vaccine-induced antibodies. Table S4 shows the percentage of β-hexosaminidase release after inhibition with different AIT sera.

**Figure 6:**
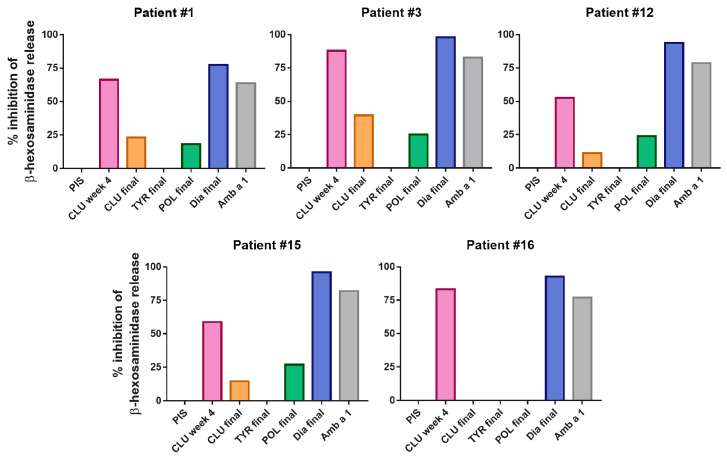
Inhibition of β-hexosaminidase release after incubation of rAmb a 1.01 with AIT-vaccine-induced rabbit antibodies. Percentages of inhibition of β-hexosaminidase release (*y*-axis) induced with 100 ng/mL rAmb a 1.01 pre-incubated with sera after CLUSTOID (CLU), TYRO-SIT (TYR), POLLINEX (POL) and Diater immunization (Dia) (*x*-axis) for RBL cells that had been sensitized with serum IgE from five ragweed-allergic patients are shown. A rabbit rAmb a 1.01-specific serum was used as a positive control. PIS, pre-immune serum.

Regarding Amb a 1-induced basophil degranulation, the best inhibition was observed upon pre-incubation with 100 ng/mL rAmb a 1.01. Figure 6 shows that the Diater-induced antibodies inhibited Amb a 1-induced basophil degranulation almost completely. The rabbit anti-CLUSTOID antibodies obtained at week 4, when the Amb a 1-specific IgG levels were high, also inhibited Amb a 1-induced basophil degranulation, although to a lesser degree than the Diater-specific antibodies (Figure 6). A low inhibition of Amb a 1-induced basophil degranulation was observed with the CLUSTOID-specific serum obtained 4 weeks after the last immunization and the POLLINEX-specific antiserum, whereas the TYRO-SIT-specific antibodies failed to inhibit Amb a 1-induced basophil degranulation (Figure 6, Table S4).

**Figure 7:**
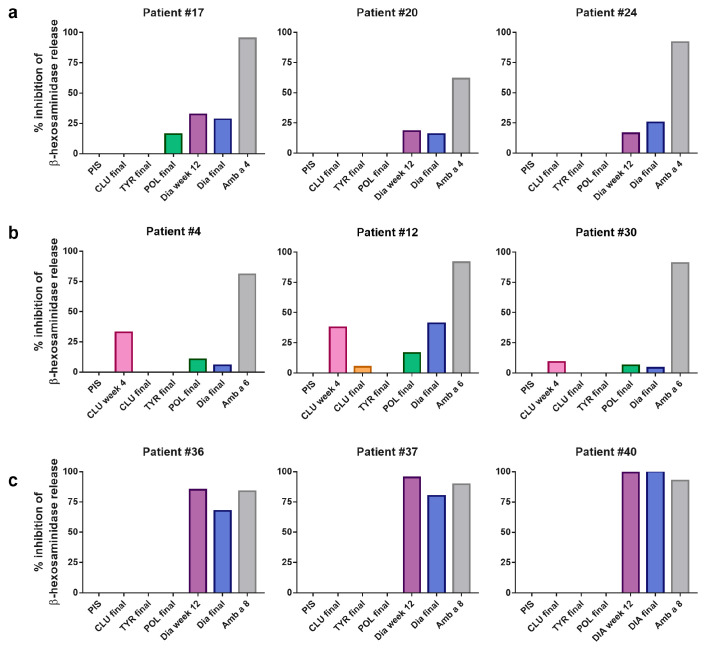
Inhibition of β-hexosaminidase release after incubation of ragweed allergens with AIT vaccine-induced sera. Percentages of inhibition of β-hexosaminidase release (*y*-axis) induced with (**a**) 100 ng/mL of rAmb a 4, (**b**) 10 ng/mL of Amb a 6 and (**c**) 10 ng/mL of Amb a 8 pre-incubated with CLUSTOID- (CLU), TYRO-SIT- (TYR), POLLINEX- (POL) and Diater-induced rabbit antibodies (Dia) (*x*-axis) when RBL cells had been sensitized with sera from three patients reactive to these allergens were tested. Rabbit allergen-specific sera were used as positive controls. PIS, pre-immune serum.

The inhibition of basophil degranulation induced with Amb a 4, Amb a 6, Amb a 8 and Amb a 11 by the AIT vaccine-induced antibodies was incomplete and highly variable (Figure 7, Table S4).

The preincubation of rAmb a 4 (100 ng/mL) with rabbit anti-Diater IS obtained at week 12 inhibited basophil degranulation between 16.5 and 32.2%, and that with the anti-Diater final IS inhibited basophil degranulation between 15.6 and 28.4% in the three patients tested (Figure 7a). The inhibition of mediator release in patient #17 upon preincubation with anti-POLLINEX final IS was 16.1%, but it was not observed in the other two patients tested.

The inhibition of rAmb a 6-dependent β-hexosaminidase release in the three patients tested was observed upon the preincubation of 10 ng/mL rAmb a 6 with anti-CLUSTOID IS obtained at week 4, ranging between 9.0 and 37.8%, with rabbit anti-Diater final IS, ranging between 4.3 and 41.1%, and with rabbit anti-POLLINEX final IS, ranging between 6.2 and 16.5% (Figure 7).

The preincubation of rAmb a 8 (10 ng/mL) with the anti-Diater IS obtained at week 12 and the final IS inhibited β-hexosaminidase release in the three patients tested, ranging between 85.1 and 99.4% and 67.5 and 100%, respectively (Figure 7c). No inhibition could be observed after the preincubation of rAmb a 8 with any of the other rabbit AIT-specific sera, whereas preincubation with 100 ng/mL of rAmb a 8 showed a lower level of inhibition with the rabbit anti-Diater IS at week 12 as well as with the anti-Diater final IS (Table S4).

The preincubation of rAmb a 11 (100 ng/mL) with the anti-Diater IS at week 8 and the final IS was able to inhibit the β-hexosaminidase release in one patient by 66.0% and 57.3%, respectively, whereas the other two tested patients did not show degranulation in response to Amb a 11 (Table S4).

## 4. Discussion

Ragweed (*Ambrosia artemisiifolia*) pollen is one of the most common causes of respiratory allergies worldwide [53]. Recent studies investigating the IgE sensitization profiles of ragweed-allergic patients showed complex IgE reactivity profiles towards the different ragweed pollen allergens [12]. Currently, the only allergy treatment with long-lasting effects is AIT [37]. AIT is highly effective when compared to pharmacological treatment and may be considered a therapeutic vaccination [37]. Accordingly, the identification of the disease-causing allergen source is an important prerequisite, and it has been shown that molecular diagnosis is not only useful for the personalization of AIT but also effective in reducing health care costs [54,55,56]. Molecular allergy diagnosis (i.e., the determination of IgE sensitizations towards the individual allergen molecules in a given allergen source) has been introduced into clinical practice [57]. However, it is known that natural allergen extracts used for the production of AIT vaccines can vary strongly regarding their allergen composition and the immunogenicity of the individual allergen molecules [46]. It has been shown that the ability of AIT vaccines to induce protective allergen-specific antibodies can be determined by immunization experiments and the consecutive measurement of the induction of protective antibodies that can inhibit allergic patients’ IgE binding to the allergens and allergen-induced basophil activation [47,48]. Likewise, it is possible to determine the effects of AIT on the induction of allergen-specific protective antibody responses by the analysis of post-AIT sera [52,58]. In this study, we investigated the antibody response of four different licensed ragweed AIT vaccines regarding their ability to induce IgG antibodies against ragweed pollen allergens and the ability of the induced antibodies to block allergic patients’ IgE binding to the allergens and allergen-induced basophil degranulation, a surrogate for immediate-type allergic symptoms.

For this purpose, rabbits were immunized with four different commercially available ragweed AIT vaccines—CLUSTOID, TYRO-SIT, POLLINEX and Diater—according to the manufacturer’s recommendations for AIT in allergic patients (Table S2). The sera were collected before the administration of the treatment, in four-week intervals, and the final serum was obtained 4 weeks after the last immunization (Figure 1). We found that the four allergen extract-based AIT vaccines induced IgG antibody responses that differed considerably regarding allergen specificity, as well as their levels and abilities to inhibit patients’ IgE binding and allergen-induced basophil degranulation.

In the two immunized rabbits, Diater induced the highest levels of ragweed pollen allergen extract-specific IgG and the highest levels of IgG antibodies against the major ragweed pollen allergen Amb a 1 (Figures S1 and S3). Diater also induced good IgG antibody production against three out of four additional ragweed allergens, which were frequently recognized by the ragweed-allergic patients tested in our study (i.e., Amb a 4, Amb a 8 and Amb a 11), whereas the levels of IgG production to Amb a 6 were lower. Diater-induced Amb a 1 antibodies remained high 4 weeks after the last immunization. In contrast, IgG antibody levels against Amb a 4, Amb a 6, Amb 8 and Amb a 11 decreased faster than Amb a 1-specific IgG. One possible explanation for the different kinetics of IgG levels towards the different allergens may be that the concentrations and/or immunogenicity of the allergens varied in the three vials containing different amounts of allergens which have been used for the Diater vaccination protocol. The different kinetics of antibody responses seem to be important because the decline of IgG antibody levels to Amb a 4, Amb a 6, Amb a 8 and Amb a 11 was associated with a reduced inhibition of patients’ IgE binding and inhibition of allergen-induced basophil activation at the time point of the final serum sampling (Figure 5, Figure 6 and Figure 7). In general, we found for all of the AIT vaccines that, at least after one course of vaccination, the levels of allergen-specific IgG antibodies were associated with the extent of inhibition of allergic patients’ IgE binding and allergen-induced basophil activation.

The four immunotherapies not only vary in their allergen content and the immunogenicity of the different allergens but also differ in the AIT administration in patients, i.e., the concentration and the number of applied doses (Figure 1, Table S2). In fact, the Amb a 1-specific IgG induction level upon immunization with Diater was comparable to that of TYRO-SIT and POLLINEX at the beginning of the immunizations (week 4), and other similarities were found between different AITs for Amb a 4, Amb a 6 and Amb a 8 at a given time point (e.g., week 8). These findings suggest that continuous immunizations with increasing doses as in the case of Diater (Table S2) could further boost the IgG response, as was shown for Amb a 1, provided that the extracts in different vials contain the respective allergen. Since it was beyond the scope of our study and would have also been difficult to perform in our animal model, we do not know how repeated vaccination schedules would affect allergen-specific IgG levels and the ability of allergen-specific IgG induced by repeated immunization to inhibit patients’ IgE binding and allergen-specific basophil activation. In fact, it is known that long-term AIT may also broaden the IgG response, especially in the IgG_4_ subclass [59] so that eventually also lower levels of allergen-specific IgG may have a strong IgE blocking activity. A previous study found that the counterpart POLLINEX vaccine for grass pollen treatment induced a modest IgG response in patients in the first year of treatment, while a significant induction of blocking IgG antibodies was only found after the second year of treatment [60]. It is therefore a limitation of our study that our results show the induction of allergen-specific IgG and allergen-specific IgE blocking antibodies only for one course of treatment with the different AIT vaccines. It is another limitation of our study that we cannot evaluate AIT mechanisms that are not based on the effects of IgE-blocking antibodies. Finally, one must bear in mind that our results were obtained by the immunization of naïve rabbits and that their immune responses may vary from those of sensitized rabbits and even more from those of allergic patients. Although evidence of the differences in the IgG response of sensitized and non-sensitized rabbits is missing, studies in children and adults have reported that pollen-sensitized patients show significantly higher IgG levels to pollen allergens than non-sensitized individuals [61,62]. However, it has been shown for the grass pollen allergy vaccine BM32 that the IgG responses of naïve rabbits were similar to those obtained in allergic patients [47,63,64]. Therefore, the rabbit immunization model may be considered very useful for the evaluation of injection immunotherapy vaccines regarding the induction of IgE-blocking antibodies.

In fact, our study seems to indicate that the Diater AIT vaccine is more effective in inducing Amb a 1-specific IgE-blocking antibodies than CLUSTOID. TYRO-SIT and POLLINEX seem to poorly induce Amb a 1-specific blocking antibodies, and there was also no relevant induction of blocking antibodies specific for Amb a 4, Amb a 6, Amb a 8 or Amb a 11. CLUSTOID was the AIT vaccine which seemed to induce blocking antibodies for Amb a 6 the best. However, it must be borne in mind that we have evaluated only one batch of the AIT vaccines and obtained data only for two rabbits. We, therefore, cannot draw conclusions for cases in which different batches are studied that are based on different natural allergen extracts, which potentially vary regarding their allergen contents and properties. Furthermore, it will be necessary to conduct immunizations in a larger number of animals to obtain representative results because there may be a variation in immune responses in different animals. Another limitation of our study that could not be evaluated in animal models and might arise in allergic patients upon AIT administration is adverse side effects. Similar to rabbits, each patient reacts differently to AIT with some reporting adverse side effects from minor to severe during the administration of AIT. In this regard, allergoids are modified to reduce the risk of serious adverse side effects and maintain low IgE levels. Thus, treating patients with Diater may induce higher allergen-specific IgG levels but may also have a higher risk of developing side effects compared to allergoids. Therefore, allergologists must decide which AIT should be prescribed considering a patient’s sensitization profile, the clinical efficacy of each AIT and the potential risk of developing adverse side effects. Furthermore, the development of blocking IgG antibodies in patients can be monitored, like as described in the rabbit model.

Although a reduced number of rabbits were immunized to minimize the amount of harm to these animals, our study could serve as a pilot study to assess the induction of blocking antibodies with different ragweed AIT vaccines using animal models. In such animal models, a large and representative number of animals need to be immunized to obtain meaningful information about the capacity of a particular AIT vaccine to induce allergen-specific IgE-blocking antibodies. The fact that different AIT vaccines can induce blocking antibodies to a varying degree against different allergens within an allergen source is interesting and may eventually open the door for personalized AIT vaccination because it is possible to identify the molecular IgE sensitization profiles of different ragweed allergic patients and prescribe AIT vaccines, which are effective in inducing blocking antibodies against the desired allergen molecules. Our study may therefore be considered useful for the selection of allergen-extract-based AIT vaccines for personalized AIT. However, due to the variability in the antibody responses of different rabbits, it will be necessary to immunize a larger number of rabbits with the AIT vaccines to reach meaningful conclusions about the ability of a particular AIT vaccine to induce blocking IgG responses against particular allergens. In fact, some studies provide support for such a concept because it has been shown that allergic patients seem to benefit more from AIT treatment with vaccines containing those allergens against which they are mainly sensitized [52,58]. Built on rabbit immunization data, as exemplified in our study, one may therefore choose AIT vaccines for patients according to their sensitization profile and monitor the induction of allergen-specific blocking antibodies by measuring their development in the course of treatment, as has been demonstrated earlier.

However, our study also shows the limitations of currently available allergen-extract-based AIT vaccines and thus emphasizes the need for molecular AIT vaccines that can induce blocking antibodies for the most relevant allergens in a given allergen source.

## 5. Conclusions

Our study showed a high level of variability in the allergen-specific IgG response in four different allergen-extract-based ragweed AITs by rabbit immunization. High allergen-specific IgG levels in the rabbit AIT-specific sera were associated with IgE inhibition in ragweed-allergic patients’ sera. Our findings highlight the importance of identifying the allergens contained in the extract-based AITs in addition to their ability to induce allergen-specific IgG antibodies capable of inhibiting IgE binding. Due to the possible variability in the immune responses induced by the AIT vaccines, further studies need to be performed using a sufficient number of rabbits to provide representative results. This information could facilitate the recommendation of ragweed AITs that address the individual IgE-sensitization profile of the ragweed-allergic patient being treated. Furthermore, the results from our study highlight the need for modern molecular AIT vaccines that induce protective antibodies against all relevant allergens in an allergen source.

## Data Availability

Data are contained within the article or Supplementary Materials.

## Supplementary Materials

The following supporting information can be downloaded at: https://www.mdpi.com/article/10.3390/vaccines12060635/s1, Figure S1: Induction of ragweed pollen allergen-specific IgG responses in rabbits by immunization with (a) CLUSTOID, (b) TYRO-SIT, (c) POLLINEX and (d) Diater. Sera from rabbits (*n* = 2) immunized with the four AITs were collected before immunization and every four weeks until final serum collection (four weeks after the last immunization). Rabbit sera were diluted from 1:100 down to 1:50,000 and tested towards ragweed pollen extract in ELISA. Shown are mean OD values corresponding to allergen-specific IgG levels (*y*-axes) at different time points (*x*-axes). IS, immune-serum; Figure S2: Titration of rabbit specific IgG antibodies towards minor ragweed pollen allergens upon immunization with four different AIT vaccines. Comparisons and time courses of specific IgG responses of rabbits (one rabbit displayed for each AIT) against rAmb a 3, rAmb a 5, rAmb 9, rAmb a 10 and rAmb a 12 upon immunization with (a) CLUSTOID, (b) TYRO-SIT, (c) POLLINEX and (d) Diater. Rabbit antisera were diluted from 1:100 down to 1:50,000. Shown are mean OD values corresponding to allergen-specific IgG levels (*y*-axes) at different time points (*x*-axes); Figure S3: IgG responses of two rabbits immunized with CLUSTOID, TYRO-SIT, POLLINEX and Diater (left to right) against (A) major and relevant ragweed pollen allergens. Sera from rabbits immunized with the four AITs (*n* = 2/AIT) were collected before immunization, every four weeks and four weeks after the last immunization. Rabbit pre-immune and immune serum were diluted 1:1000. Shown are mean OD values corresponding to allergen-specific IgG levels (*y*-axes) at different time points (*x*-axes). IgG responses of two rabbits immunized with CLUSTOID, TYRO-SIT, POLLINEX and Diater (left to right) against (B) minor ragweed pollen allergens. Sera from rabbits immunized with the four AITs (*n* = 2/AIT) were collected before immunization, every four weeks and four weeks after the last immunization. Rabbit pre-immune and immune serum were diluted 1:1000. Shown are mean OD values corresponding to allergen-specific IgG levels (*y*-axes) at different time points (*x*-axes); Table S1: IgE reactivity of patients towards major and minor ragweed pollen allergens tested in ELISA (*n* = 50). The optical density (OD 405 nm) displayed in the table correspond to the levels of IgE antibodies. Values considered positive for each allergen are highlighted in grey; Table S2: The immunization protocols of four commercially available AIT vaccines to be used in ragweed-allergic patients. Below is the dosage according to the manufacturer’s recommendation for immunization with CLUSTOID Cluster-Allergoid (ROXALL Medizin, Vienna, Austria), TYRO-SIT and POLLINEX Quattro Plus (Bencard Allergie GmbH, Munich, Germany) and Diater Depot (DIATER, Madrid, Spain); Table S3: Inhibition of patients’ IgE binding to ragweed pollen extract and allergens with rabbit AIT-specific sera. Rabbit PIS (pre-immune serum), IS (immune serum) and final IS were used to inhibit IgE binding of patients in ELISA; Table S4: Inhibition of the β-hexosaminidase release to rAmb a 1.01, rAmb a 4, rAmb a 6, rAmb a 8 and rAmb a 11 with rabbit AIT-specific sera in mediator release assay. Rabbit pre-immune (PIS) and allergen-specific serum were used as controls. IS, immune serum.
