# Supplementary material for: Heterogenous Induction of Blocking Antibodies against Ragweed Allergen Molecules by Allergen Extract-Based Immunotherapy Vaccines
Source: Vaccines (Basel). 2024 Jun 7;12(6):635. doi: 10.3390/vaccines12060635 (PMC11209568; doi:10.3390/vaccines12060635)
Supplement: Supplementary file 1 [file vaccines-12-00635-s001.zip › Supplementary Figure S1.pdf]

Supplementary Figure S1

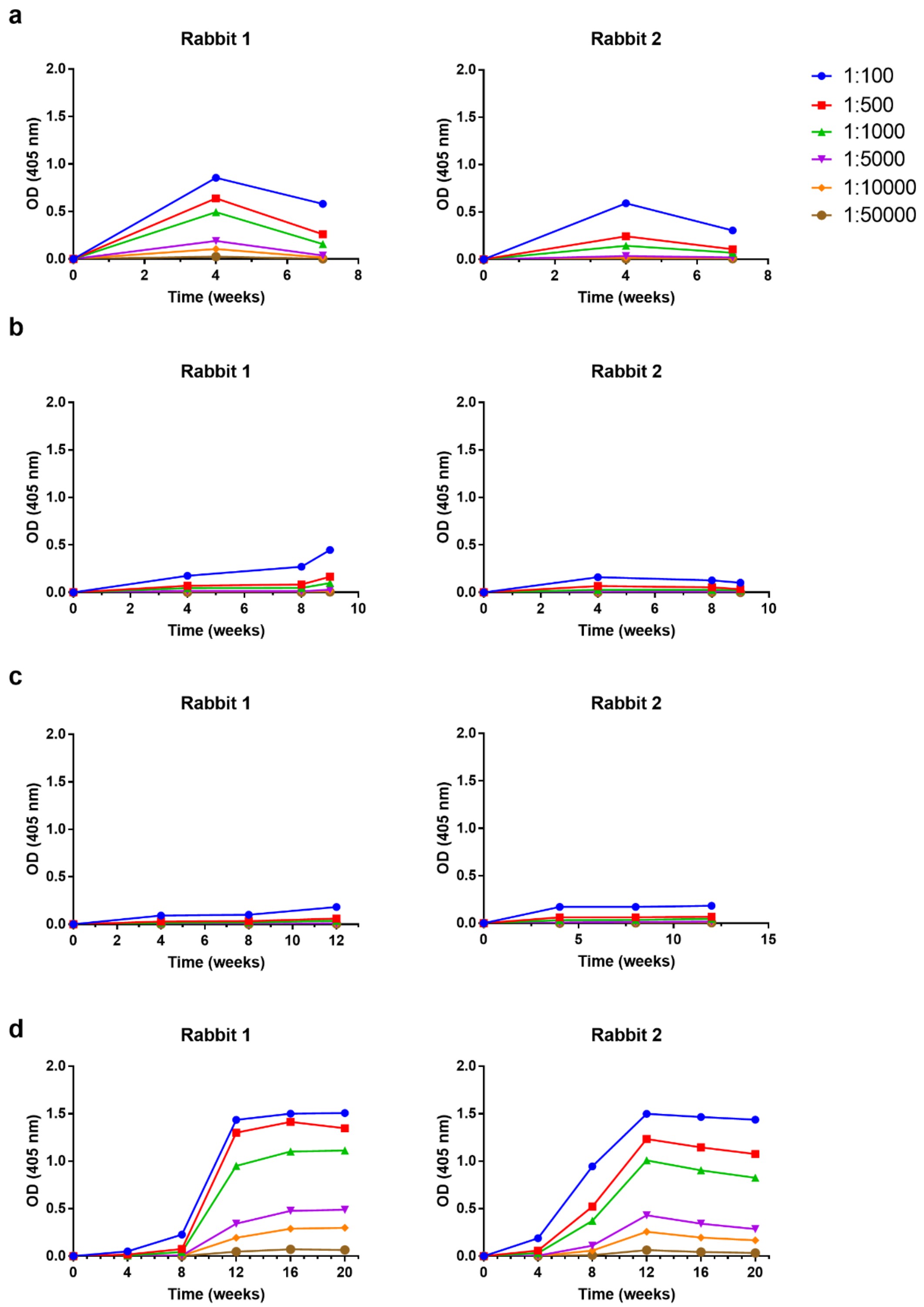

Figure S1. Induction of ragweed pollen allergen-specific IgG responses in rabbits by immunization with (**a**) CLUSTOID, (**b**) TYRO-SIT, (**c**) POLLINEX and (**d**) Diater. Sera from rabbits (n = 2) immunized with the four AITs were collected before immunization and every four weeks until final serum collection (four weeks after the last immunization). Rabbit sera were diluted from 1:100 down to 1:50,000 and tested towards ragweed pollen extract in ELISA. Shown are mean OD values corresponding to allergen-specific IgG levels (*y*-axes) at different time points (*x*-axes). IS, immune-serum.
