# Supplementary material for: Heterogenous Induction of Blocking Antibodies against Ragweed Allergen Molecules by Allergen Extract-Based Immunotherapy Vaccines
Source: Vaccines (Basel). 2024 Jun 7;12(6):635. doi: 10.3390/vaccines12060635 (PMC11209568; doi:10.3390/vaccines12060635)
Supplement: Supplementary file 1 [file vaccines-12-00635-s001.zip › Supplementary Figure S2.pdf]

Supplementary Figure S2

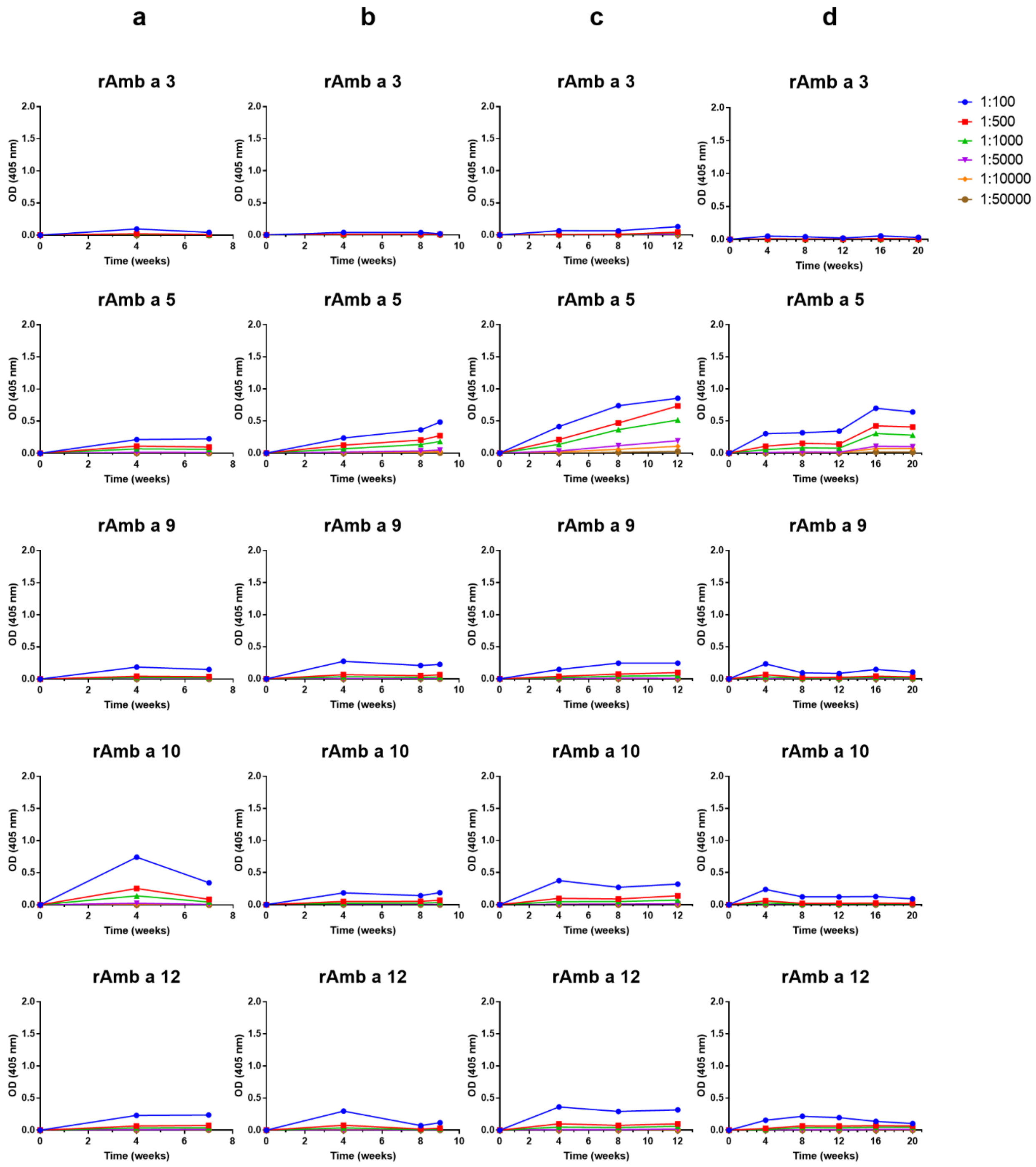

Figure S2. Titration of rabbit specific IgG antibodies towards minor ragweed pollen allergens upon immunization with four different AIT vaccines. Comparisons and time courses of specific IgG responses of rabbits (one rabbit displayed for each AIT) against rAmb a 3, rAmb a 5, rAmb 9, rAmb a 10 and rAmb a 12 upon immunization with (**a**) CLUSTOID, (**b**) TYRO-SIT, (**c**) POLLINEX and (**d**) Diater. Rabbit antisera were diluted from 1:100 down to 1:50,000. Shown are mean OD values corresponding to allergen-specific IgG levels (*y*-axes) at different time points (*x*-axes).
