# Supplementary material for: Heterogenous Induction of Blocking Antibodies against Ragweed Allergen Molecules by Allergen Extract-Based Immunotherapy Vaccines
Source: Vaccines (Basel). 2024 Jun 7;12(6):635. doi: 10.3390/vaccines12060635 (PMC11209568; doi:10.3390/vaccines12060635)
Supplement: Supplementary file 1 [file vaccines-12-00635-s001.zip › Supplementary Figure S3.pdf]

Supplementary Figure S3

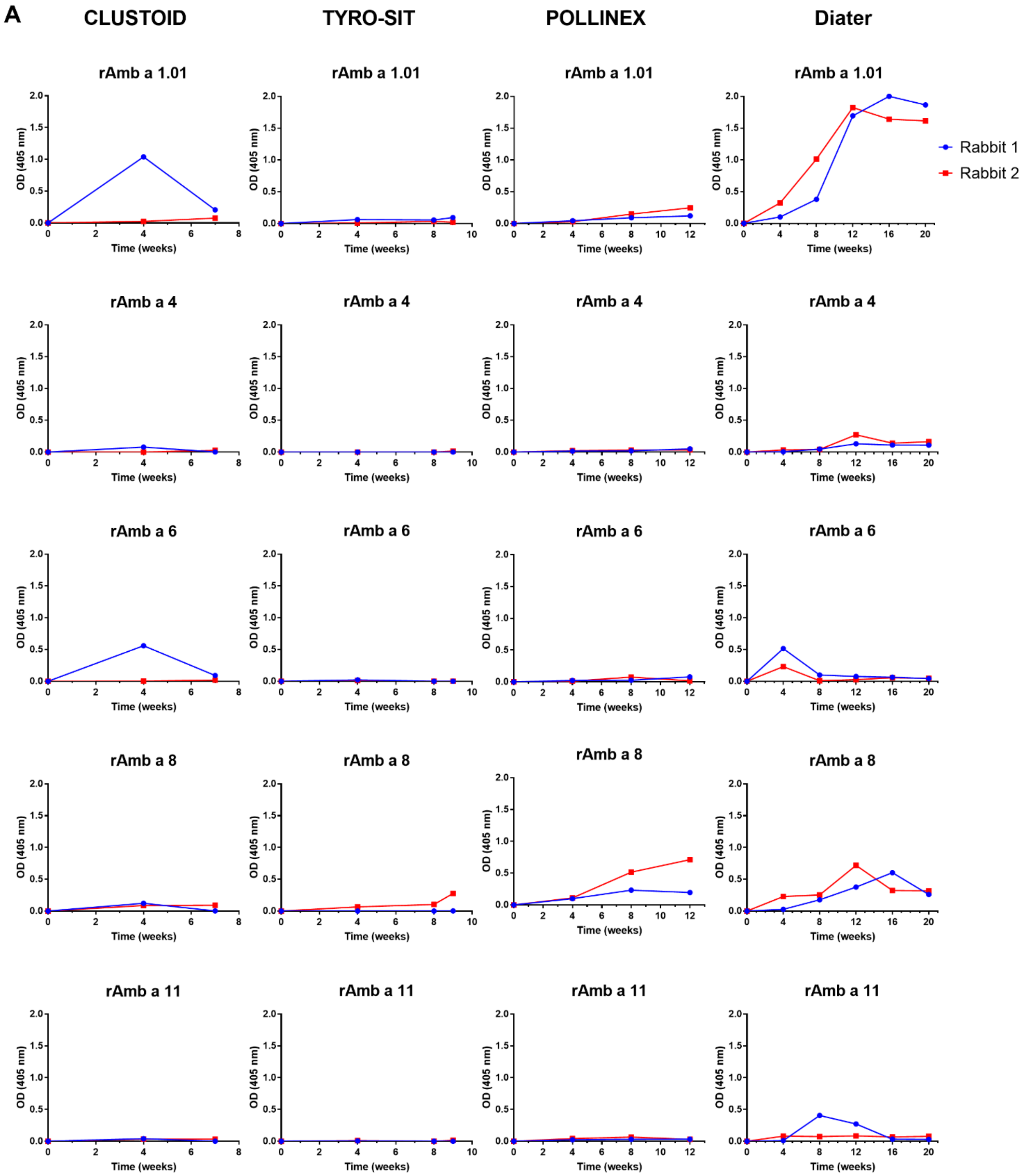

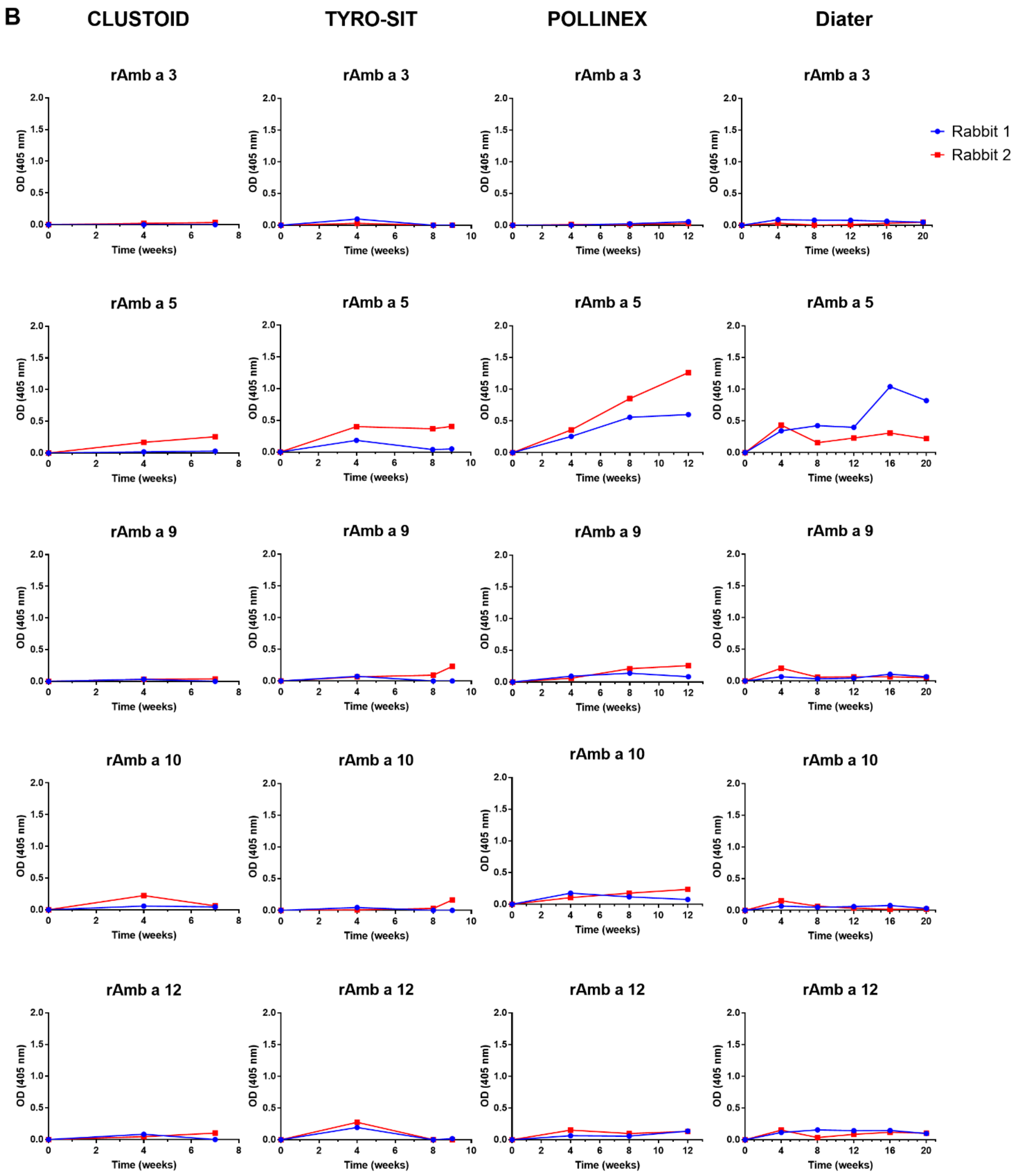

Figure S3. IgG responses of two rabbits immunized with CLUSTOID, TYRO-SIT, POLLINEX and Diater (left to right) against (**A**) major and relevant ragweed pollen allergens. Sera from rabbits immunized with the four AITs (n = 2/AIT) were collected before immunization, every four weeks and four weeks after the last immunization. Rabbit pre-immune and immune serum were diluted 1:1000. Shown are mean OD values corresponding to allergen-specific IgG levels (*y*-axes) at different time points (*x*-axes). IgG responses of two rabbits immunized with CLUSTOID, TYRO-SIT, POLLINEX and Diater (left to right) against (**B**) minor ragweed pollen allergens. Sera from rabbits immunized with the four AITs (n = 2/AIT) were collected before immunization, every four weeks and four

weeks after the last immunization. Rabbit pre-immune and immune serum were diluted 1:1000. Shown are mean OD values corresponding to allergen-specific IgG levels (*y*-axes) at different time points (*x*-axes).
