# Supplementary material for: Heterogenous Induction of Blocking Antibodies against Ragweed Allergen Molecules by Allergen Extract-Based Immunotherapy Vaccines
Source: Vaccines (Basel). 2024 Jun 7;12(6):635. doi: 10.3390/vaccines12060635 (PMC11209568; doi:10.3390/vaccines12060635)
Supplement: Supplementary file 1 [file vaccines-12-00635-s001.zip › Supplementary Table S1.pdf]

Table S1. IgE reactivity of patients towards major and minor ragweed pollen allergens tested in ELISA (n = 50). The optical density (OD 405 nm) displayed in the table correspond to the levels of IgE antibodies. Values considered positive for each allergen are highlighted in grey.

Patient code	IgE reactivity to recombinant ragweed pollen allergens (OD)										
	**Amb a 1.01**	**Amb a 1.03**	**Amb a 3**	**Amb a 4**	**Amb a 5**	**Amb a 6**	**Amb a 8**	**Amb a 9**	**Amb a 10**	**Amb a 11**	**Amb a 12**
**#1**	1.72	1.54	n/a	1.26	0.10	0.06	0.87	0.08	0.15	0.10	0.19
**#2**	2.96	3.06	0.16	0.37	0.10	0.23	0.06	0.09	0.08	0.22	0.53
**#3**	0.84	1.31	0.11	1.41	0.08	0.05	0.06	0.06	0.07	0.09	0.15
**#4**	1.83	2.34	0.20	0.08	0.12	3.41	0.07	0.09	0.12	0.33	0.33
**#5**	1.11	1.25	0.08	0.15	1.01	0.06	0.05	0.06	0.08	0.40	0.15
**#6**	1.28	1.31	n/a	0.11	0.10	0.06	0.08	0.10	0.18	0.25	0.42
**#7**	1.92	1.98	n/a	0.13	0.18	0.18	0.11	0.15	0.23	0.47	0.44
**#8**	1.47	1.76	n/a	0.21	0.16	0.97	0.10	0.12	0.24	0.26	0.59
**#9**	1.48	1.88	n/a	0.10	0.11	0.07	0.52	0.08	0.12	0.29	0.34
**#10**	1.74	1.99	n/a	0.13	0.14	0.24	0.15	0.24	0.21	0.44	0.36
**#11**	2.28	2.39	n/a	0.14	0.14	0.07	0.10	0.09	0.16	0.36	0.31
**#12**	2.62	2.48	0.11	0.07	0.09	2.45	0.06	0.06	0.09	0.18	0.15
**#13**	2.93	2.73	0.09	0.07	0.09	0.06	0.66	0.07	0.10	0.29	0.18
**#14**	2.90	2.94	0.13	0.08	0.12	0.07	0.43	0.09	0.15	0.35	0.23
**#15**	2.48	2.81	0.11	0.06	0.08	0.06	0.06	0.06	0.06	0.17	0.18
**#16**	1.94	2.31	0.10	0.07	0.12	0.06	0.07	0.06	0.08	0.21	0.97
**#17**	0.28	1.25	n/a	3.27	0.11	0.07	0.08	0.08	0.09	0.25	0.19
**#18**	0.72	0.92	n/a	1.32	0.14	0.95	0.13	0.08	0.19	0.27	0.33
**#19**	1.88	1.84	n/a	3.10	0.11	3.32	0.07	0.08	0.10	0.30	0.18
**#20**	2.46	2.24	n/a	1.88	0.35	0.06	0.12	0.09	0.26	1.33	0.41
**#21**	1.16	1.57	n/a	0.89	0.13	0.07	0.08	0.08	0.16	0.66	0.20
**#22**	1.59	1.50	0.13	0.95	0.14	0.07	0.10	0.12	0.13	0.35	0.61
**#23**	1.07	0.84	0.11	1.24	0.08	0.06	0.06	0.08	0.13	0.15	0.30
**#24**	1.37	1.78	0.20	1.71	0.18	3.25	0.06	0.07	0.08	0.17	0.20
**#25**	2.40	2.30	0.17	0.23	0.15	1.54	0.09	0.11	0.15	1.09	0.73
**#26**	0.64	0.66	0.10	0.07	0.10	1.89	0.19	0.08	0.08	0.19	0.12
**#27**	0.69	0.50	n/a	0.10	0.11	1.30	0.08	0.11	0.27	0.22	0.81
**#28**	2.08	2.10	n/a	0.14	0.13	1.41	0.09	0.09	0.13	0.25	0.27
**#29**	2.25	2.20	0.12	0.08	0.13	2.53	0.20	0.18	0.09	0.31	0.51

**#30**	2.15	2.27	0.12	0.08	0.35	3.50	0.22	0.06	0.18	0.20	0.25
**#31**	2.29	2.26	0.09	0.09	0.08	1.64	0.06	0.06	0.16	0.39	0.33
**#32**	2.39	2.64	0.10	0.07	0.08	3.65	0.05	0.07	0.13	0.31	0.30
**#33**	2.99	2.80	n/a	0.10	0.11	0.08	1.06	0.08	0.19	0.77	0.37
**#34**	2.46	2.67	n/a	0.11	0.09	0.07	0.98	0.09	0.11	0.46	0.17
**#35**	2.40	2.66	0.18	0.11	0.18	0.08	1.63	0.12	0.15	0.23	0.32
**#36**	3.22	3.33	0.08	0.07	0.08	0.05	1.96	0.06	0.08	0.13	0.12
**#37**	2.22	2.36	0.11	0.85	0.08	0.07	1.34	0.11	0.10	0.23	0.22
**#38**	1.26	1.07	n/a	0.12	0.20	0.67	0.43	0.08	0.28	0.98	0.41
**#39**	2.12	2.23	n/a	0.14	0.15	0.74	0.83	0.08	0.14	0.59	0.27
**#40**	0.88	1.34	n/a	0.11	0.10	0.07	1.37	0.13	0.16	0.38	0.26
**#41**	0.08	0.06	n/a	0.11	0.12	3.60	0.86	0.09	0.14	0.28	0.22
**#42**	1.43	1.21	n/a	0.15	0.14	0.07	0.10	0.11	0.47	1.05	0.65
**#43**	0.95	1.01	n/a	0.08	0.12	0.71	0.07	0.09	0.18	1.02	0.25
**#44**	0.38	0.24	0.11	0.08	0.07	0.08	0.06	0.06	0.08	1.55	0.22
**#45**	2.05	1.94	n/a	0.11	0.25	0.06	0.08	0.08	0.11	1.00	0.48
**#46**	1.37	1.26	n/a	0.11	0.11	0.06	0.07	0.07	0.10	1.05	0.24
**#47**	0.19	0.17	n/a	0.29	0.12	0.07	0.08	0.09	0.33	1.31	0.36
**#48**	0.40	0.29	n/a	0.18	0.15	0.07	0.09	0.09	0.13	1.33	0.55
**#49**	2.41	1.86	n/a	0.17	0.10	0.08	0.07	0.09	0.30	0.91	1.42
**#50**	0.64	0.62	0.21	0.10	0.10	0.08	0.11	0.07	0.11	0.88	0.19

n/a, not available; cut-off value was defined for each allergen as mean OD plus three times the standard deviation of three sera from ragweed non-allergic patients.
