# Supplementary material for: Heterogenous Induction of Blocking Antibodies against Ragweed Allergen Molecules by Allergen Extract-Based Immunotherapy Vaccines
Source: Vaccines (Basel). 2024 Jun 7;12(6):635. doi: 10.3390/vaccines12060635 (PMC11209568; doi:10.3390/vaccines12060635)
Supplement: Supplementary file 1 [file vaccines-12-00635-s001.zip › Supplementary Table S2.pdf]

Table S2. The immunization protocols of four commercially available AIT vaccines to be used in ragweed-allergic patients. Below is the dosage according to the manufacturer's recommendation for immunization with CLUSTOID Cluster-Allergoid (ROXALL Medizin, Vienna, Austria), TYRO-SIT and POLLINEX Quattro Plus (Bencard Allergie GmbH, Munich, Germany) and Diater Depot (DIATER, Madrid, Spain).

Week no.	AIT immunization dosage concentration (volume), vial no.			
	CLUSTOID	TYRO-SIT	POLLINEX	Diater
**Week 0**	2000 TU (0.2 mL), vial 1<br>5000 TU (0.5 mL), vial 1	2000 TU (0.1 mL), vial 1	300 SU (1 mL), vial 1	1/100 (0.1 mL), vial 1
**Week 1**	5000 TU (0.5 mL), vial 1	6000 TU (0.3 mL), vial 1		1/100 (0.2 mL), vial 1
**Week 2**	5000 TU (0.5 mL), vial 1	10.000 TU (0.5 mL), vial 1	800 SU (1 mL), vial 2	1/100 (0.4 mL), vial 1
**Week 3**	5000 TU (0.5 mL), vial 1	16.000 TU (0.1 mL), vial 2		1/100 (0.8 mL), vial 1
**Week 4**		48.000 TU (0.3 mL), vial 2	2000 SU (1 mL), vial 3	1/10 (0.1 mL), vial 2
**Week 5**		80.000 TU (0.5 mL), vial 2		1/10 (0.2 mL), vial 2
**Week 6**				1/10 (0.4 mL), vial 2
**Week 7**				1/10 (0.8 mL), vial 2
**Week 8**			2000 SU (1 mL), vial 3	1/1 (0.1 mL), vial 3
**Week 9**				1/1 (0.2 mL), vial 3
**Week 10**				1/1 (0.4 mL), vial 3
**Week 11**				1/1 (0.6 mL), vial 3
**Week 12**				1/1 (0.8 mL), vial 3
**Week 13**				
**Week 14**				
**Week 15**				
**Week 16**				1/1 (0.8 mL), vial 3
