# Supplementary material for: Heterogenous Induction of Blocking Antibodies against Ragweed Allergen Molecules by Allergen Extract-Based Immunotherapy Vaccines
Source: Vaccines (Basel). 2024 Jun 7;12(6):635. doi: 10.3390/vaccines12060635 (PMC11209568; doi:10.3390/vaccines12060635)
Supplement: Supplementary file 1 [file vaccines-12-00635-s001.zip › Supplementary Table S4.pdf]

Table S4. Inhibition of the β-hexosaminidase release to rAmb a 1.01, rAmb a 4, rAmb a 6, rAmb a 8 and rAmb a 11 with rabbit AIT-specific sera in mediator release assay. Rabbit pre-immune (PIS) and allergen-specific serum were used as controls. IS, immune serum.

Allergen	Pat ID	Conc. ng/ml	% β-hexosaminidase release upon preincubation with rabbit PIS and IS							
			PIS	CLUSTOID week 4	CLUSTOID final	TYRO-SIT final	POLLINEX final	Diater week 8/12	Diater final	Allergen specific
rAmb a 1.01	#1	100	68.5	19.9	45.3	73.3	48.3	n/a	13.4	21.3
		10	33.8	9.2	32.5	42.0	32.9	n/a	11.9	23.8
		1	–	–	–	–	–	n/a	–	–
	#3	100	46.2	5.6	27.9	51.7	34.5	n/a	0.9	7.9
		10	13.0	0.8	12.3	21.9	15.0	n/a	1.2	6.6
		1	–	–	–	–	–	n/a	–	–
	#12	100	97.0	46.1	86.2	108.6	73.8	n/a	6.0	20.8
		10	47.5	18.0	38.8	66.4	65.2	n/a	8.2	24.1
		1	–	–	–	–	–	n/a	–	–
	#15	100	76.5	31.5	65.3	85.0	55.9	n/a	3.0	13.8
		10	34.2	6.9	31.4	40.0	37.4	n/a	4.4	16.4
		1	–	–	–	–	–	n/a	–	–
	#16	100	35.0	5.9	35.8	51.5	0.0	n/a	2.6	8.0
		10	–	–	–	–	–	n/a	–	–
		1	–	–	–	–	–	n/a	–	–
rAmb a 4	#17	100	40.8	n/a	47.8	52.0	34.2	27.7	29.2	1.9
		10	33.9	n/a	33.9	38.3	33.9	29.3	32.2	9.7
		1	28.3	n/a	34.7	44.8	30.5	7.8	15.1	16.1
	#20	100	17.3	n/a	30.9	26.7	24.9	14.2	14.6	6.6
		10	12.6	n/a	24.7	23.7	31.3	3.3	2.3	18.1
		1	-	n/a	-	-	-	-	-	-
	#24	100	45.6	n/a	51.3	48.6	58.1	38.1	34.0	3.7
		10	40.7	n/a	42.7	39.6	45.7	35.7	32.1	8.1
		1	34.0	n/a	39.7	36.6	44.6	20.6	13.0	16.7
rAmb a 6	#4	100	62.1	48.5	68.3	63.0	30.9	n/a	32.8	5.7
		10	38.3	25.7	40.1	39.0	34.3	n/a	36.2	7.4
		1	32.4	17.1	33.9	35.0	28.9	n/a	26.5	7.1
	#12	100	61.8	51.6	64.9	71.5	38.2	n/a	26.3	5.8
		10	46.0	28.6	43.6	47.1	38.4	n/a	27.1	3.8
		1	30.3	17.3	34.2	29.9	36.6	n/a	22.5	2.6
	#30	100	79.4	67.0	89.7	86.8	55.7	n/a	46.3	3.1
		10	65.6	53.6	69.5	60.6	61.5	n/a	56.5	5.3
		1	59.4	41.3	60.4	64.1	56.7	n/a	50.4	8.5
rAmb a 8	#36	100	36.4	n/a	39.4	44.7	38.5	28.4	28.7	5.3
		10	28.5	n/a	33.5	33.9	30.6	4.2	9.2	4.6
		1	–	n/a	–	–	–	*n/a*	–	–
	#37	100	41.0	n/a	41.0	45.8	48.9	24.9	19.6	0.4
		10	30.1	n/a	36.1	42.3	42.7	2.4	11.5	2.4
		1	–	n/a	–	–	–	*n/a*	–	–
	#40	100	35.1	n/a	44.2	43.3	41.0	22.5	16.2	2.3
		10	19.3	n/a	32.7	33.3	33.6	3.2	5.4	4.5
		1	–	n/a	–	–	–	–	–	–
rAmb a 11	#25	100	38.4	n/a	56.7	56.6	52.1	13.0	16.4	26.9
		10	–	n/a	–	–	–	–	–	–
		1	–	n/a	–	–	–	–	–	–
	#43 #44	100	–	n/a	–	–	–	–	–	–
		10	–	n/a	–	–	–	–	–	–
		1	–	n/a	–	–	–	–	–	–

n/a, not applicable; –, % degranulation ≤ negative control (huRBL cells preincubated with patient serum).
